# Association between Two Resistin Gene Polymorphisms and Metabolic Syndrome in Jilin, Northeast China: A Case-Control Study

**DOI:** 10.1155/2017/1638769

**Published:** 2017-12-14

**Authors:** Yingli Fu, Yaqin Yu, Yanhua Wu, Yueyue You, Yangyu Zhang, Changgui Kou

**Affiliations:** ^1^Department of Epidemiology and Biostatistics, School of Public Health, Jilin University, Changchun, China; ^2^Division of Clinical Epidemiology, First Hospital of Jilin University, Changchun, China

## Abstract

Metabolic syndrome (MetS) is a significant health care problem worldwide and is characterized by increased fasting glucose and obesity. Resistin is a protein hormone produced both by adipocytes and immunocompetent cells, including those residing in adipose tissue, and is believed to modulate glucose tolerance and insulin action. This study examined the association of resistin gene polymorphisms, rs1862513 and rs3745368, and related haplotypes with the development of metabolic syndrome in a Han Chinese population. This case-control study was performed on 3792 subjects, including 1771 MetS cases and 2021 healthy controls from the Jilin province of China. Metabolic syndrome was defined according to the criteria of the International Diabetes Federation (IDF). Logistic regression analysis was used to estimate the relationship between gene polymorphism and MetS. Our results showed that there were no significant associations between MetS and the genotype distributions in four kinds of inheritance models, allele frequencies, and related haplotypes of resistin gene polymorphisms rs1862513 and rs3745368 (all *p* values > 0.05). Based on our study findings, we concluded that mutations in resistin genes are not associated with the presence of MetS in a Han Chinese population from Jilin province in China.

## 1. Introduction

Metabolic syndrome (MetS) is defined by a combination of interconnected physiological, biochemical, clinical, and metabolic factors. It is characterized by a cluster of risk factors, including elevated blood pressure, increased fasting glucose, central obesity, and dyslipidemia (defined by increased triglycerides and reduced high-density lipoprotein cholesterol), as defined by the International Diabetes Federation (IDF) [1].

MetS confers an increased risk of diabetes mellitus, cardiovascular disease, stroke, myocardial infarction, and all-cause mortality. It is well recognized as a growing public health and clinical challenge worldwide, especially for low- and middle-income countries [1–3]. The prevalence of MetS around the world ranges from 10% to 84% [4]. The age-standardized prevalence of MetS among Chinese adults is 10.5%, according to IDF criteria and data from the China Health and Nutrition Surveys (CHNS) in 2009 [5]. Due to the high health risks associated with MetS, the understanding of the precise pathogenesis of this metabolic disorder is the subject of intense investigation [6].

Resistin is a protein hormone produced both by adipocytes and immunocompetent cells, including those residing in adipose tissue [7]. Some evidence [8–10] suggests that resistin modulates glucose tolerance and insulin action, thereby playing a role in the pathogenesis of obesity and insulin resistance in humans. RETN, the gene coding for human resistin, is located on chromosome 19p13.3.6 [7]. The length of the RETN prepeptide in humans is 108 amino acids [8]. Taking into consideration that diabetes mellitus and obesity are major characteristics of MetS, the resistin gene was considered as a potential candidate gene for MetS in this study.

Up to 70% of the variation in circulating resistin levels can be explained by genetic factors, and several single-nucleotide polymorphisms in the *RETN* gene have been described so far [11, 12]. One of the most frequently studied polymorphisms, *RETN* rs1862513, was reported to be associated with the regulation of *RETN* gene expression and serum resistin level [13–15]. Several studies have also associated the *RETN* rs1862513 polymorphism with obesity [16–18], insulin sensitivity [19], type 2 diabetes [15], and cerebrovascular disease [20]. As single-nucleotide polymorphisms (SNPs) in the 3′-untranslated region (3'UTR) of genes can affect gene expression and disease susceptibility [21], the rs3745368 SNP in the 3'UTR of the resistin gene might have an influence on resistin gene expression and thus influences the risk for the development of diabetes and hypertension [22].

The role of resistin in MetS remains controversial. Some studies showed that resistin levels correlated with obesity and diabetes [23, 24], while others failed to observe any correlation of resistin levels with metabolic markers [25, 26]. Meanwhile, several studies have shown that ethnicity plays an important role in this inconsistency [27, 28].

The primary goal of our study was to estimate the impact of *RETN* polymorphisms rs1862513 and rs3745368 and their haplotypes on MetS and its individual components in a Northeastern Chinese population.

## 2. Materials and Methods

### 2.1. Study Population

A multistage stratified cluster sampling process was used to select the participants from nine areas of Jilin Province, Northeast China, in a community-based survey conducted in 2012. There were four stages of sampling. In the first stage, the province was stratified by the nine regions corresponding to the administrative areas that are largely responsible for health care delivery. Secondly, four districts or counties were randomly selected from each of the nine regions using probability proportional to size (PPS) sampling. In the third stage, four or five communities were selected from both the rural and urban strata of each district or county using PPS. In the last stage, one resident was randomly selected from each household of each community. More detailed information on the sampling process can be found in previous publications [29, 30]. In this case-control study, frequency matching was used for the sampling. According to IDF criteria, 5702 Han Chinese individuals in the study were diagnosed with MetS. Among these individuals, there were 3792 participants who were without missing values on the necessary information, 1771 were randomly selected as cases from these 3792 participants, and 2021 subjects without MetS were recruited from the same source population as controls. We selected this subsample to optimize the genotyping costs and have sufficient power to detect statistically significant differences. The average age and the percent of male were matched within case and control groups.

In this study, MetS was diagnosed using the 2009 IDF criteria [31] that subjects must meet, that is, three or more of the following five criteria are required to be diagnosed with MetS: (a) elevated waist circumference ≥ 85 cm in males and ≥80 cm in females; (b) elevated triglycerides ≥ 150 mg/dL (1.7 mmol/L) or drug treatment for elevated triglycerides; (c) high-density lipoprotein cholesterol (HDL-C) < 40 mg/dL (1.0 mmol/L) in males and <50 mg/dL (1.3 mmol/L) in females or drug treatment for reduced HDL-C; (d) elevated blood pressure with systolic ≥ 130 and/or diastolic ≥ 85 mm Hg or antihypertensive drug treatment; and (e) elevated fasting glucose ≥ 100 mg/dL or drug treatment of elevated glucose. These criteria were generated by an IDF consensus group in 2004, with representatives from the organizations that had generated the previous definitions and members from all IDF regions. It allows for comparative long-term studies and is applicable to populations around the world [27, 32].

Demographic information (age, sex) and lifestyle factors (alcohol consumption and smoking status) were obtained by a self-report questionnaire. Body mass index (BMI), waist circumference, hip circumference, systolic blood pressure (SBP), diastolic blood pressure (DBP), and heart rate were measured by physical examination. Alcohol consumption was categorized into three levels according to self-reported frequency of drinking per week: normal drinker, often drinker, and never drinker. Subjects who drank over three times per week were categorized as often drinkers; those who drank more than one time but less than three times per week were categorized as normal drinkers, and those who drank less than one time per week during their lifetime were categorized as never drinkers. The smoking status was categorized into current smoker, former smoker, and never smoker according to the self-reported lifetime number of cigarettes and number of smoking days. Individuals who had smoked at least 100 cigarettes in their lifetime and were smoking during the time of the survey were defined as current smokers; individuals who had smoked at least 100 cigarettes but gave up smoking before the time of the survey were defined as former smokers, and individuals who had smoked fewer than 100 cigarettes during their lifetime were defined as never smoked. BMI was calculated as weight (kg)/height (m^2^). Height and weight were measured in subjects standing straight and wearing light clothing without shoes. The waistline and hip circumferences were measured separately at the level of the individual's umbilicus and the maximum protrusion of the gluteal muscles, respectively. The SBP and DBP were recorded with an average of two measurements in a sitting position after a 10-minute rest period with a mercury sphygmomanometer. Enzymatic methods were used to measure the total cholesterol (TC), triglycerides (TG), HDL-C, and low-density lipoprotein cholesterol (LDL-C) from blood lipids in a central laboratory. 5 mL blood samples were collected from all subjects in the morning after an overnight fast (at least 8 hours) and transported to the laboratory under refrigeration, and all four indexes mentioned above were assessed within 12 hours. Fasting blood glucose (FBG) was measured by a Bai Ankang fingertip blood glucose monitor (Bayer, Leverkusen, Germany) using fingertip blood samples. Both the demographic and clinical information were obtained at the same interview and test. More details on the methods have been reported in previous publications [33].

The ethics committee of the School of Public Health, Jilin University, approved our study and all subjects signed written informed consent before participating in this study.

### 2.2. DNA Extraction and Genotyping

According to previous studies [13, 15, 22], SNPs rs1862513 and rs3745368 were selected by the Haploview program (https://www.ncbi.nlm.nih.gov/gene/) to identify the association between *RETN* gene polymorphism and MetS. The minor allele frequencies of these two SNPs were greater than 0.05 in Han Chinese population. A commercial DNA extraction kit (JiuNa Biotech Company, Hangzhou, China) was used to extract genomic DNA from peripheral blood lymphocyte samples (5 mL). Assay design 3.1 software (Sequenom Inc., San Diego, CA) was used for designing the primers in polymerase chain reaction (PCR) for all the SNPs. SNP genotyping was determined using matrix-assisted laser desorption/ionization time of flight mass spectrometry (MALDI-TOF-MS). SNP genotyping reactions were performed in a 384-well Spectro-CHIP using a MassARRAY nanodispenser (Sequenom Inc.).

### 2.3. Statistical Analyses

The data analyses were conducted using STATA/IC (Version 14.2; Texas, USA) and the online SNP Stats (https://www.snpstats.net/snpstats/start.htm?) program. Demographic characteristics and MetS associated biochemical parameters were compared for subjects with and without MetS using the Pearson's chi-square (*χ*^2^) and Student's *t*-test. The genotype distribution and allele frequency of the SNPs were compared between cases and controls using *χ*^2^ test. For each SNP, a Hardy-Weinberg disequilibrium (HWD) test was conducted in the control group. Logistic regression was used to calculate the odds ratios (OR) and 95% confidence interval (95% CI). SNP Stats was used to examine the potential associations between *RETN* gene haplotypes and risk of MetS. All tests were two sided, and statistical significance was considered as a *p* value < 0.05.

## 3. Results

### 3.1. Demographic Characteristics and Biochemical Features of Study Population

The average age of the case and control groups was 49.49 ± 0.23 and 49.45 ± 0.21 years, respectively. The percent of the male of the case and control groups was 49.9% and 50.2%, respectively. The level of high-density lipoprotein cholesterol (HLD-C) was lower in the case group than that in the control group (*p* < 0.001), as expected. In contrast, the levels of other biochemical parameters were higher in the case group compared with the control group (Table 1).

### 3.2. Distributions of Genotype and Allele Frequencies in the Case and Control Groups

The results of the HWD test for the control group showed that the two *RETN* SNPs (rs1862513, 3745368) were in accordance with the HWD. As shown in Table 2, the frequencies of the genotypes and alleles of the two SNPs were not significantly different between the case and control groups, and there were no significant associations between both rs1862513 and rs3745368 gene polymorphisms and the risk of MetS after controlling for demographic and lifestyle factors in our results.

### 3.3. Distributions of Genotypes in the Case and Control Groups in Four Inheritance Models

Table 3 shows the adjusted odds ratios of genotypes in four inheritance models (codominant, dominant, recessive, and overdominant); results revealed no significant association between MetS risk and the two investigated single-nucleotide variants (rs1862513 and rs3745368) of the *RETN* gene (*p* > 0.05).

### 3.4. Association between Haplotypes and the Risk of MetS

Haplotype distributions were also performed and there was no significant difference between subjects with and without MetS (all *p* > 0.05) (Table 4).

## 4. Discussion

This case-control study was performed on 3792 subjects, including 1771 MetS cases and 2021 healthy controls, to explore the association between two resistin gene (rs1862513 and rs3745368) polymorphisms and MetS among Han Chinese population of Jilin, Northeast China. The major findings of this study were that there was no significant association between MetS and the genotype distributions in four types of inheritance models, allele frequencies, and related haplotypes of resistin gene rs1862513 and rs3745368 polymorphisms.

For rs1862513 gene polymorphism, the results in our study were consistent with previous reports in Thais [34] and in nondiabetic Caucasians [35] that resistin 5' variant rs1862513 probably do not influence susceptibility to MetS or any other metabolic feature including glucose, lipids, waist circumference, and blood pressure. But the results are still debatable. Some studies found that the GG genotype, or G-allele, of rs1862513 was correlated with increased prevalence of MetS [36–38]. And a study conducted by Boumaiza and his colleagues in Tunisian volunteers showed that the rs1862513 polymorphism was associated with risk of MetS components, such as obesity, higher waist circumference, higher BMI, and increased TC and LDL-C levels [7].

Regarding the associations between the rs3745368 polymorphism and MetS, early studies reported varying results [7, 39–41]. The presence of the G/A genotype was more frequent in the MetS group in Spanish subjects [39], and the presence of the A-allele was associated with metabolic markers in Mexican subjects [41]. By contrast, the polymorphism of rs3745368 was not associated with MetS risk in Tunisian [7] as well as Polish women [40], and these results were confirmed by our study.

One explanation of the discrepancy could be that the distribution of the polymorphism of interest appears to be quite different in distinct ethnic populations, and these genetic differences may contribute to varying prevalence rates of MetS among ethnic groups. Furthermore, the criteria for MetS' diagnosis could be a probable reason for the discrepancy. In our study, we used IDF criteria to diagnose MetS, but some other studies defined MetS according to the National Cholesterol Education Program Adult Treatment Panel III (NECP-ATPIII) [37] criteria. Compared with other criteria, the IDF definition requires obesity, but not necessarily insulin resistance. Also, different populations, ethnicities, and nationalities have a different distribution of norms for body weight and waist circumference, which could influence the results as well. Additionally, lifestyle, such as physical activity, sedentary lifestyle, and dietary habits, probably is an important factor that contributes to inconsistent results, as this factor affects the development of MetS [42, 43]. For example, among the Northeast Chinese people, most food preparations contain excessive salt, and this may be a risk factor for some MetS components, such as hypertension [44, 45]. Sedentary lifestyle, levels of work, and physical activity may also influence the development of MetS through BMI as a mediator [43].

This study presents novel data and findings that may have important implications for assessing MetS risk in the Han Chinese population of Northeast China. However, this study also has some limitations. First of all, MetS is the result of several combined factors; yet in this study, we only analyzed the correlation between *RETN* polymorphisms and MetS, thereby ignoring environmental factors and gene-environment interactions, which should be examined further. In addition, only two *RETN* polymorphisms were screened in this study, and further studies may be required to explore more thoroughly the possible role of resistin in the development of MetS. Furthermore, genotyping errors may introduce exposure identification bias, although genetic case-control studies suffer less with classification biases than classic epidemiologic studies [46]. Genotyping errors are common due to the presence of unknown SNPs in the neighboring nucleotides of a target SNP. Each method chosen can undergo misidentification of the SNP and error in the genotyping that obviously create a bias for analysis [47]. In our study, we did not evaluate the scale of genotyping errors. In summary, the search for potential gene candidates that may influence the development of MetS requires further investigation.

## Figures and Tables

**Table 1:** Demographic and biochemical parameters of study population.

Parameters	Case (*n* = 1771)	Control (*n* = 2021)	*t*/*χ*^2^	*p*
Age (year)	49.49 ± 0.23	49.45 ± 0.21	0.115	0.907
Gender				
Male	884 (49.9)	1015 (50.2)	0.036	0.850
Female	887 (50.1)	1006 (49.8)		
BMI (kg/m^2^)	27.21 ± 0.07	21.90 ± 0.06	54.533	<0.001^∗^
WC (cm)	91.28 ± 0.19	75.02 ± 0.16	65.743	<0.001^∗^
HC (cm)	99.73 ± 0.15	90.74 ± 0.13	45.067	<0.001^∗^
HR (beat/min)	79.06 ± 0.26	74.26 ± 0.25	13.230	<0.001^∗^
SBP (mm Hg)	144.78 ± 0.45	120.22 ± 0.35	42.720	<0.001^∗^
DBP (mm Hg)	87.93 ± 0.26	74.61 ± 0.21	39.805	<0.001^∗^
TG (mg/dL)	3.23 ± 0.06	1.09 ± 0.01	34.188	<0.001^∗^
TC (mg/dL)	5.26 ± 0.03	4.72 ± 0.02	15.272	<0.001^∗^
LDL-C (mg/dL)	3.08 ± 0.02	2.84 ± 0.02	8.488	<0.001^∗^
HDL-C (mg/dL)	1.16 ± 0.01	1.62 ± 0.01	−43.347	<0.001^∗^
Fasting glucose (mg/dL)	6.64 ± 2.44	4.85 ± 0.98	28.918	<0.001^∗^

Data are given as mean ± SD or frequency (% subjects); *p* values were analyzed using Student's *t*-test or nonparametric test. ^∗^A value of *p* < 0.05 was considered statistically significant. BMI: body mass index; WC: waist circumference; HC: hip circumference; HR: heart rate; SBP: systolic blood pressure; DBP: diastolic blood pressure; TG: triglyceride; TC: total cholesterol; LDL-C: low-density lipoprotein cholesterol; HDL-C: high-density lipoprotein cholesterol.

**Table 2:** Distributions of genotypes/alleles and the risk estimates for the variant genotypes/alleles.

SNP	Genotype	Cases (%)	Controls (%)	Crude OR (95% CI)	*p*	Adjusted OR^∗^ (95% CI)	*p*
rs1862513	CC	191 (10.78)	218 (10.79)	1	0.906	1	0.505
	GG	800 (45.17)	918 (45.42)	0.99 (0.80–1.23)		0.98 (0.76–1.25)	
	CG	780 (44.04)	885 (43.79)	1.01 (0.81–1.25)		1.00 (0.78–1.27)	
	C-allele	1162 (32.81)	1321 (32.68)	1	0.908	1	0.727
	G-allele	2380 (76.19)	2721 (67.32)	0.99 (0.90–1.10)		0.982 (0.89–1.09)	

rs37445368	GG	1304 (73.63)	1471 (72.79)	1	0.541	1	0.536
	AA	33 (1.86)	37 (1.83)	1.01 (0.63–1.62)		0.98 (0.58–1.66)	
	GA	434 (24.51)	513 (25.38)	0.95 (0.82–1.11)		0.95 (0.81–1.12)	
	G-allele	3042 (85.88)	3455 (85.48)	1	0.615	1	0.588
	A-allele	500 (14.12)	587 (14.52)	0.97 (0.85–1.10)		0.96 (0.84–1.11)	

OR: odds ratio; CI: confidence interval. ^∗^Adjusted for age, sex, smoking status, and alcohol consumption.

**Table 3:** Genotype distribution by different inheritance models and odds ratio estimate.

SNP	Genotype	Inheritance model	Case	Control	Adjusted OR (95% CI)^∗^	*p*
rs1862513	C/C	Codominant	191 (10.8%)	218 (10.8%)	1	0.96
	G/G		800 (45.2%)	918 (45.4%)	0.98 (0.79–1.21)	
	G/C		780 (44%)	885 (43.8%)	0.99 (0.80–1.23)	
	C/C	Dominant	191 (10.8%)	218 (10.8%)	1	0.88
	G/G-C/G		1580 (89.2%)	1803 (89.2%)	0.98 (0.80–1.21)	
	C/C-G/G	Recessive	991 (56%)	1136 (56.2%)	1	0.86
	G/C		780 (44%)	885 (43.8%)	1.01 (0.89–1.15)	
	C/C-G/C	Overdominant	971 (54.8%)	1103 (54.6%)	1	0.78
	G/G		800 (45.2%)	918 (45.4%)	0.98 (0.86–1.12)	

rs3745368	G/G	Codominant	1304 (73.6%)	1471 (72.8%)	1	0.84
	A/A		33 (1.9%)	37 (1.8%)	0.97 (0.60–1.57)	
	A/G		434 (24.5%)	513 (25.4%)	0.96 (0.82–1.11)	
	G/G	Dominant	1304 (73.6%)	1471 (72.8%)	1	0.56
	A/A-A/G		467 (26.4%)	550 (27.2%)	0.96 (0.83–1.11)	
	G/G-A/A	Recessive	1337 (75.5%)	1508 (74.6%)	1	0.56
	A/G		434 (24.5%)	513 (25.4%)	0.96 (0.83–1.11)	
	G/G-A/G	Overdominant	1738 (98.1%)	1984 (98.2%)	1	0.95
	A/A		33 (1.9%)	37 (1.8%)	0.98 (0.61–1.59)	

OR: odds ratio; CI: confidence interval. ^∗^Adjusted for age, sex, smoking status, and alcohol status.

**Table 4:** Associations between *RETN* haplotypes and risk of MetS.

rs1862513	rs3745368	Frequency			Adjusted OR (95%)	*p*
		Total	Case	Control		
G	G	0.6400	0.6395	0.6404	1	
C	G	0.2167	0.2193	0.2144	1.03 (0.92–1.16)	0.60
C	A	0.1107	0.1088	0.1124	0.97 (0.84–1.13)	0.73
G	A	0.0326	0.0324	0.0328	0.96 (0.72–1.30)	0.81
